# Abnormal High-sensitivity C-reactive Protein is Associated with an Increased Risk of Cardiovascular Disease and Renal Dysfunction among Patients Diagnosed with Type 2 Diabetes Mellitus in Palestine

**DOI:** 10.1900/RDS.2022.18.27

**Published:** 2022-03-31

**Authors:** Mohammed Husham Khattab, Moyad Jamal Shahwan, Nageeb Abdul Galil Mohamed Hassan, Ammar Abdulrahman Jairoun

**Affiliations:** 1 Department of Clinical Sciences, College of Pharmacy and Health Sciences, Ajman University, Ajman, United Arab Emirates,; 2 Center of Medical and Bio-allied Health Sciences Research, Ajman University, Ajman, United Arab Emirates,; 3 Consumer Product Safety Section, Public Health and Safety Department, Dubai Municipality, Dubai, United Arab Emirates.

**Keywords:** type 2 diabetes, c-reactive protein, hsCRP, inflammation, glomerular filtration rate, renal function

## Abstract

**OBJECTIVE:**

In this study, we aimed to evaluate the prevalence of high sensitivity C-reactive protein (hsCRP) as an inflammatory mediator and its association with renal function and other biochemical markers in patients with type 2 diabetes mellitus.

**METHODS:**

We carried out a cross-sectional study at private healthcare center. We included 453 patients (48.6% males and 51.4% females) with type 2 diabetes mellitus. We obtained sociodemographic, clinical, and laboratory data from patient medical records. We carried out statistical analysis to ascertain associations between parameters.

**RESULTS:**

The overall risk of cardiovascular disease (hsCRP > 1 mg/L) among the study participants was 27.2%. Age, gender, body mass index, fasting blood glucose and serum creatinine were significantly associated with risk of cardiovascular disease (hsCRP > 1 mg/L) whereas estimated glomerular filtration rate, vitamin B12, calcium, sodium and metformin users were negatively associated with the hsCRP.

**CONCLUSIONS:**

We found a significant positive association of elevated level of C-reactive protein with type 2 diabetes mellitus. Moreover, additional to increased cardiovascular disease risk, hsCRP also seems to be a major inflammatory risk marker indicating renal function loss.

## 1 Introduction

Type 2 diabetes mellitus (T2DM) is a well-known risk factor for various cardiovascular diseases and yjpl,’ has been estimated to increase the incidence of life- threatening events by 2-fold [1,2]. Moreover, over one- third of T2DM patients remain undiagnosed. According to the most recent report by International Diabetes Federation (IDF) diabetes atlas (9^th^ edition), one out of every 2 adults with diabetes remains undiagnosed. Worldwide the number of these undiagnosed cases is estimated to be over 232 million [3].

The risk of developing heart diseases in diabetes patients has been reported to be more prevalent among females compared to males [4]. The pathophysiology and severity of glucose disorders has been associated with abnormalities in inflammatory markers [5]. Substantial evidence now relates the pathogenesis of diabetes to several inflammatory markers acting as intermediate factors, which link T2DM to several coexisting inflammatory conditions [6]. C-reactive protein (CRP) is one of the general markers indicating systemic inflammation. It is an acute phase protein that originates from the liver and has high sensitivity in response to chronic and acute systemic inflammation [7]. This low-grade chronic inflammation is now being linked with an elevation in CRP levels. Thus, the rise in CRP level may be another reason behind the development and manifestation of T2DM despite the mechanism not being clear [8]. According to one metaanalysis and systematic review, the risk of diabetes was reported to be much higher in those having elevated levels of hsCRP [9]. Furthermore, hsCRP can act as an important predictor for various cardiovascular diseases especially myocardial infarction, even though levels of this inflammatory marker may vary within populations by age, obesity status, and gender [10]. CRP exhibits high stability and frozen blood samples easily can be stored for the long-term while maintaining stability; in addition, its relatively cheap and accurate standardized tests have made studying this specific protein more accessible [11]. Studies suggest that measurement of these inflammatory markers predicts the risk of development and progression of diabetes, and hence, suggests implementation of early lifestyle interventions for these individuals [12,13].

Previous studies show some contradictory results. Authors of one meta-analysis found there was a direct relationship between hsCRP levels and an increased risk of diabetes. However, another meta-analysis found no such association, and the level of hsCRP was not considered as an independent risk factor for risk of diabetes development [14,15]. The level of hsCRP usually is seen to be higher among individuals experiencing diabetic nephropathy. Additionally, an elevated level of hsCRP is considered as to be the largest mortality factor in patients with cardiovascular diseases and chronic renal failure who requires dialysis. Moreover, because renal function loss and renal diseases are linked with an elevated hsCRP level, this can be helpful in addressing the risk factor for the mentioned conditions [16].

Therefore, the present study was designed to estimate the prevalence of abnormal inflammatory mediators and the magnitude of the relationships among hsCRP levels, renal function, and other biochemical markers in patients with T2DM.

## 2 Methods

### 2.1 Subjects and materials

We conducted a cross-sectional study in a healthcare center in Ramallah district, Palestine. The participants were outpatients, consecutively recruited by specialist diabetes physician, at regular follow-up visits. The systematic sample consisted of 453 patients diagnosed with T2DM, of whom 220 were males and 233 were females. The study occurred from March 2019 through October 2019; the study was approved by the health and ethics committee of the health center, and all participants gave their informed consent in accordance with the Declaration of Helsinki [17]. Relevant sociodemographic, clinical and laboratory data were obtained from the medical records of the patients including age, gender, height, weight, BMI, HbA1c, FBS, medications history, hsCRP, creatinine, e-GFR, albumin, vitamins, and other selected variables.

### 2.2 Definitions

Having an HbA1c > 6.5% was used as the diagnosis criterion for T2DM [18]. Patients were considered to have a risk of cardiovascular disease if there was an hsCRP > 1 mg/L, a level defined as an abnormal CRP. An hsCRP level of lower than 1.0 mg/L indicates low risk of CVD. These cut off values were based on Cleveland Clinic recommendations as part of their blood tests to determine risk of coronary artery disease [19,20].

### 2.3 Inclusion and exclusion criteria

Patients diagnosed with T2DM at age 18 and above were included, having a regular monitoring check-up including clinical laboratory data in 2019. The study excluded persons with a chronic disease medical history and long-term treatment other than forT2DM, as well as patients with renal, cardiovascular, or neurological abnormality. In addition, children, pregnant women, and patients with type 1 diabetes were excluded.

### 2.4 Data analysis

Statistical analysis was carried out using the Statistical Package for the Social Sciences (SPSS, version 23). Frequencies and percentages were used to summarize the occurrence of variables. Graphical representations were provided for all relevant variables. Statistical differences in proportions of variables were assessed using chi-square and Fisher’s Exact Test. Univariate and multivariate logistic regression models were performed to ascertain the statistically significant associations among hsCRP, renal function, and various biochemical parameters among patients with T2DM, and other related risk factors. For variable selection and model building, we used stepwise procedure. We used p-values< 0.05 and 95% confidence intervals as the criteria to make decisions regarding statistical significance.

## 3 Results

**Table 1** shows the demographic and socio-economic characteristics of the study participants. A total of 453 subjects with diabetes were included in this study. Among these, 48.6% (n=220) were male and 51.4% (n=233) were female. The age of the participants was distributed as follows: 30% were 28-51 years old, 45.3%were 52-62 years old, and 24.7% > 62 years old. Of the participants, 29.6% were single and 70.4% were married. About 82 (18.1%) of the participants had an elementary school education, 178 (39.3%) had a high school education, and 193 (42.6%) had a bachelor’s degree. About three-fourths of the participants, (77%) were non-smokers and one-third (32.7%) were not physically active.

**Table 1.** Frequency table for demographic, socio-economic characteristics and medications history of the participants (n=453)

Demographic characteristics	Groups	Frequency (%)
**Gender**	Male	220 (48.6%)
	Female	233 (51.4%)
**Age group (years)**	28-51	136 (30%)
	52-62	205 (45.3%)
	> 62	112 (24.7%)
**Marital status**	Single	134 (29.6%)
	Married	319 (70.4%)
**Educational level**	Elementary	82 (18.1%)
	High school	178 (39.3%)
	Bachelor’s degree	193 (42.6%)
**Smoking Status**	No	350 (77.3%)
	Yes	103 (22.7%)
**Physical activity**	Yes	148 (32.7%)
	No	305 (67.3%)
**Taking metformin**	Yes	90 (19.9%)
	No	363 (80.1%)
**Taking sulfonylurea**	Yes	12 (2.6%)
	No	441 (97.4%)
**Taking insulin**	Yes	58 (12.8%)
	No	395 (87.2%)
**Taking statin**	Yes	42 (9.3%)
	No	411 (90.7%)

### 3.1 Medication history, clinical and biochemical markers of the study participants

**Table 1** shows the medication history of the study subjects. Of these, 19.9% (n=90), 2.6% (n=12), 12.8% (n=58), and 9.3% (n=42) were metformin, sulfonylurea, insulin, and statin users respectively. **Table 2** shows participants’ clinical and biochemical markers.

**Table 2.** Clinical and biochemical characteristics of the participants (n=453)

Parameters	All patients (n=453)		
	Mean ± S. D	Median	Range
**Age(years)**	54.47± 10.6	65	75 -23
**Height(cm)**	166.5± 10.15	163	182 - 150
**Weight (Kg)**	81.28± 13	81	118 - 54
**BMI (kg/m^2^)**	29.35± 4.49	28.84	46.3 - 22.5
**HbA1c (%)**	7.78± 1.26	7.6	11.1 - 5.3
**FBG (mg/dl)**	158.8± 50.4	149	337 -70
**Creatinine (gmol/l)**	0.78± 0.3	0.80	2.2 - 0.40
**BUN (mg/dl)**	14.8± 6.8	12	45 - 8
**e-GFR (mg/dl)**	255.6± 101.9	220.3	564 - 63.7
**Albumin (g/dL)**	4.27± 0.55	4.1	9.60 - 2.9
**Vitamin D (ng/mL)**	37.12± 18.7	38	391 - 3.9
**Vitamin B12 (pg/mL)**	271.37± 117.7	243	820 - 8.1
**Calcium (mg/dl)**	8.9± 0.66	8.9	10.5 - 3.3
**Potassium (mmo/L)**	4.56± 0.5	4.6	5.9 - 0.90
**Sodium (mmo/L)**	143.9± 5.47	144	151 - 130

**Abbreviations:** BMI, Body mass index; HbA1c, hemoglobin A1c; FBG, fasting blood glucose; BUN, Blood urea nitrogen; e-GFR, Estimated glomerular filtration rate; SD, standard deviation

### 3.2 Prevalence of the abnormal inflammatory mediator (hsCRP) in patients with type 2 diabetes mellitus

The overall risk of cardiovascular disease (hsCRP > 1 mg/L) among study participants was 27.2% [95%CI: 23-31.^3^]. Among males, 34 (19.5%) [95%CI: 14.^3^-^24^.^8^.^3^] were at risk of cardiovascular disease as were 80 females (34.3%) [95%CI: 28.^2^-40.^5^]. **Table 3** shows the prevalence of risk of cardiovascular disease by demographic variables. The risk of cardiovascular disease (hsCRP > 1 mg/L) was significantly associated with marital status (p<0.001), educational level (p<0.001) and smoking status (p<0.001).

**Table 3.** Prevalence of abnormal C-reactive protein (CRP) among men and women by demographic and lifestyle variables

Risk for cardiovascular disease CRP (> 1 mg/L)				
	All	Male	Female	p-value
	(n=453)	(n=220)	(n=233)	
**Total**	123(27.2%)	43 (34.9%)	80 (65.04 %)	< 0.001
**Age**				
28-51	15 (11%)	9 (60%)	6 (40%)	
52-62	65 (31.7%)	21 (32.3%)	44 (67.7%)	0.093
> 62	43 (38.4%)	13 (30.2%)	30 (69.8%)	
**Marital status**				
Single	26 (19.4%)	0	26 (100%)	< 0.001
Married	97 (30.4%)	43 (44.3%)	54 (55.7%)	
**Educational level**				
Elementary	32 (39%)	0	32 (100%)	
High school	66 (37.1%)	24 (36.4%)	42 (63.6%)	< 0.001
Bachelor’s degree	25 (13%)	19 (76%)	6 (24%)	
**Smoking status**				
Non-smoker	99 (28.3%)	20 (20.2%)	79 (79.8%)	< 0.001
Smoker	24 (23.3%)	23 (95.8%)	1 (4.2%)	
**Physical activity**				
Yes	23 (15.5%)	6 (26.1%)	17 (73.9%)	
No	100 (32.8%)	37 (37%)	63 (63%)	0.322

A p-value less than 0.05 was considered statistically significant.

### 3.3 Bivariate correlation between the abnormal inflammatory mediator (hsCRP) and biochemical parameters in patients with type 2 diabetes mellitus

There was a statistically significant positive correlation between abnormal hsCRP and BMI (r=0.125, p=0.008), FBG (r=0.183, p< .001), serum creatinine (r=0.129, p=0.006), BUN (r=0.227, p<0.001) and statin use (r=0.216, p<0.001) (**Table 4**). On the other hand, we found a significant negative correlation between abnormal hsCRP and e-GFR (r=-0.119, p=0.012), vitamin B12 (r=-0.120, p=0.010), calcium (r=-0.227, p< 0.001), sodium (r=-0.154, p=0.001). and metformin use (r=- 0.180, p<0.001).

**Table 4.** Bivariate correlations between abnormal inflammatory indicators and biochemical characteristics of the participants (n=453)

Biochemical characteristics	Abnormal CRP	
	r	P-Value
**BMI(kg/m2)**	0.125	0.008
**HbA1c (%)**	0.078	0.096
**FBG (mg/dl)**	0.183	0.000
**Creatinine (gmol/l)**	0.129	0.006
**BUN (mg/dl)**	0.227	0.000
**e-GFR (mg/dl)**	- 0.119	0.012
**Albumin (g/dL)**	- 0.051	0.275
**Vitamin D (ng/mL)**	- 0.052	0.270
**Vitamin B12 (pg/mL)**	- 0.120	0.010
**Calcium (mg/dl)**	- 0.227	0.000
**Potassium (mmo/L)**	0.037	0.430
**Sodium (mmo/L)**	- 0.154	0.001
**Taking metformin**	- 0.180	0.000
**Taking sulfonylurea**	- 0.039	0.409
**Taking insulin**	0.048	0.305
**Taking statin**	0.216	0.000

**Abbreviations:** BMI, Body mass index; HbA1c, hemoglobin A1c; FBG, fasting blood glucose; BUN, Blood urea nitrogen; e-GFR, estimated glomerular filtration rate; r, Pearson correlation coefficient; p-values less than 0.05 were considered statistically significant.

### 3.4 Univariate and Multivariate analysis for the factors associated with abnormal hsCRP (hsCRP > 1 mg/L) in patients with type 2 diabetes mellitus

From the univariate analysis, increased risk of cardiovascular disease (hsCRP > 1 mg/L) was significantly associated with serum creatinine (OR=2.41; 95% CI 1.26-4.6), BUN (OR=1.07; 95% CI 1.04-1.10), e-GFR (OR=0.997; 95% CI 0.995-0.999), albumin (OR=0.592; 95% CI 0.359-0.974), vitamin B12 (OR=0.997; 95% CI 0.996-0.999), calcium (OR=0.415; 95% CI 0.284-0.608) and sodium (OR=0.940; 95% CI 0.905-0.976) (**Table 5**).

**Table 5.** Simple logistic regression analysis of factors associated with abnormal CRP

Factors				Abnormal CRP > 1 mg/dl				
		Unadjusted				Adjusted		
	OR	95% CI		P. value	OR	95% CI		P. value
**Creatinine (µmol/l)**	2.41	1.26	4.60	0.008	3.88	1.47	10.20	0.006
**BUN (mg/dl)**	1.07	1.04	1.10	0.000	1.095	1.048	1.145	0.000
**e-GFR (mg/dl)**	0.997	0.995	0.999	0.012	0.995	0.992	0.998	0.001
**Albumin (g/dL)**	0.592	0.359	0.974	0.039	0.522	0.287	0.949	0.003
**Vitamin D (ng/mL)**	0.992	0.978	1.006	0.287	1.001	0.991	1.011	0.905
**VitaminB12(pg/mL)**	0.997	0.996	0.999	0.011	0.999	0.997	1.001	0.327
**Calcium (mg/dl)**	0.415	0.284	0.608	0.000	0.404	0.226	0.722	0.002
**Potassium (mmo/L)**	1.187	0.776	1.814	0.429	1.424	0.859	2.361	0.170
**Sodium (mmo/L)**	0.940	0.905	0.976	0.001	0.962	0.921	1.006	0.087

**Notes:** Adjustment for metformin use, sulfonylurea use, insulin use, statin use, smoking, physical activity; P values less than 0.05 were considered statistically significant. **Abbreviations:** OR, odds ratio; CI, confidence interval; BUN, Blood urea nitrogen; e-GFR, Estimated glomerular filtration rate.

After stepwise multivariate analysis, the only variables that remained associated significantly with risk of cardiovascular disease (hsCRP > 1 mg/L) were age, FBG, BUN, calcium, albumin, potassium, and statin use. If age increased by one year, then the odds of having hsCRP > 1 mg/dl increased by 3%. If FBG increased by 1 mg/dl, then the odds of having hsCRP > 1 mg/dl increased by 1.3 %. If BUN increased by 1 mg/dl, then the odds of having hsCRP > 1 mg/dl increased by 7.6%. If calcium increased by 1 mg/dl, then the odds of having hsCRP> 1 mg/dl decreased by 67.6%. If albumin increased by 1 g/dL, then the odds of having hsCRP> 1 mg/dl decreased by 60.3%. If potassium increased by 1 mmo/L, then the odds of having hsCRP> 1 mg/ dl increased by 73.4%. Moreover, the odds of having hsCRP> 1 mg/dl was significantly higher in patients who used statins (**Table 6**).

**Table 6.** Multivariate logistic regression analysis for factors associated with abnormal CRP

Factors		Abnormal CRP > 1 mg/dl		
	OR	95% CI		P.value
**Age years**	1.059	1.029	1.089	0.000
**FBG (mg/dl)**	1.013	1.006	1.020	0.000
**BUN (mg/dl)**	1.076	1.019	1.137	0.009
**Calcium (mg/dl)**	0.324	0.202	0.519	0.000
**Albumin (g/dL)**	0.397	0.204	0.772	0.007
**Potassium (mmo/L)**	1.734	1.011	2.973	0.046
**Sodium (mmo/L)**	0.959	0.913	1.007	0.090
**Statin Users**	3.655	1.606	8.320	0.002

**Abbreviations:** OR, odds ratio; CI, confidence interval; HbA1c, hemoglobin A1c; FBG, fasting blood glucose; BUN, Blood urea nitrogen; e-GFR, estimated glomerular filtration rate; *p* values less than 0.05 were considered statistically significant.

## 4 Discussion

In our study population, after full adjustment for and exclusion of confounders, the analysis showed that the concentration of hsCRP was positively associated with an increased risk of T2DM. We included 453patients with T2DM. Elevated hsCRP levels were shown to be more common among the older age groups (>50 years). Higher risk was also more apparent when hsCRP was combined with high BMI and obesity. CRP is a known inflammatory indicator that is commonly detected in blood in higher concentrations among individuals experiencing inflammatory conditions as well as certain conditions such as T2DM and cardiovascular diseases [21]. Several studies have evaluated the relationship and confirmed the positive association between hsCRP and an increased risk of T2DM. These studies suggest that the hsCRP marker can be used as an independent predictor for the incidence of T2DM, which can help in the detection of development and progression of this condition [22,23]. However, in another study of 293 patients with diabetes, the association between hsCRP and diabetes was minor and negligible [24]. In our study population, the overall prevalence of abnormal hsCRP was 27.2%. Similar findings were reported by King et al in which over half (51.5%) of the subjects with diabetes had abnormal hsCRP levels, whereas higher tendency of CRP abnormalities was associated with poor glycemic control and elevated levels of HbA1c [25]. When it comes to gender, females had a higher percentage of (hsCRP > 1 mg/L) than males, which puts them at higher risk for cardiovascular diseases. Overall, 34.3% of females and 19.5% of males had levels of hsCRP above the normal range. These findings align with results of other studies where the abnormal hsCRP readings were significantly linked with T2DM in both genders. However, a more prominent association was found among females than males [23,26]. Another study in Europe found that hsCRP was independently higher among women with diabetes. Additionally, the author of that study suggested that the association between the two parameters was strongly influenced by gender. A possible explanation for this variability in women can be due to the difference in body composition and fat distribution as well as the levels of sex hormones. Also, the levels of inflammatory markers are strongly associated with the extent of adiposity percentage, and hence, a greater association is seen in females than in males [27].

Regarding patient age, we found that age was significantly associated with increased risk of cardiovascular diseases among persons experiencing T2DM; therefore, when age increased by 1 year, the odds of having hsCRP > 1 mg/dl increased by 3%, thus leading to higher risk of heart disease. Likewise, in a European study, women between the ages of 55 and 74 years had an increased risk of T2DM in relation to high plasma CRP [28]. The majority of T2DM cases are accompanied by a cluster of additional risk factors, including obesity, with relatively high body mass index (BMI) and insulin resistance [29]. The relationship between obesity (as measured in BMI) and hsCRP was examined throughout our study, and obesity was significantly associated with higher levels of hsCRP. In contrast, other studies have reported that the association between hsCRP and T2DM was eliminated after controlling BMI and/or waist circumference [30,31]. However, in our study, despite obesity being a major contributor of hsCRP level, we found that the abnormal hsCRP was not limited to obesity alone. Higher fasting blood glucose levels also were positively associated with plasma CRP; for instance, if FBG increased by 1 mg/dl, then the odds of having hsCRP > 1 mg/dl increased by 1.3%. Earlier studies view that the pro-inflammatory effect of these markers can significantly contribute to the development of serious cardiovascular events even among healthy middle- aged adults who are having higher CRP levels than the normal. These findings suggest that the higher hsCRP level can impose a much higher risk when combined with diabetes [32,33]. We further hypothesized that higher levels of plasma CRP were associated with abnormalities in renal function among T2DM patients. It is worth mentioning that we found serum creatinine, BUN and e-GFR all to be inter correlated to some extent with elevated hsCRP levels. There was a statistically significant positive correlation between abnormal hsCRP and serum creatinine, as well as between hsCRP and BUN; however, we found a negative correlation between abnormal hsCRP and e-GFR. Data relating the association between the role of low- grade inflammation such as CRP on the abnormalities in renal function are limited. The exact mechanism behind this relationship remains to be clarified. Weiss et al’s small sample of T2DM subjects showed a possible explanation was due to raised serum levels of monocyte activation marker, neopterin, and pentosidine which has led to doubling the level of serum creatinine among these subjects. Additionally, in the same study, the serum CRP was significantly higher among those experiencing diabetic nephropathy [34]. Also, elevated body fat can initiate an early inflammation process, which in turn, may lead to a loss in renal function due to the exposure of the kidneys to glomerular hyperfiltration [35]. On the other hand, when it comes to medications, we found a statistically significant negative correlation between abnormal hsCRP and metformin use. Levels of glucose and insulin, which are activated by the AMP-kinase pathway, seem to be linked with the levels of hsCRP. Reduced levels of hsCRP are reported among metformin users, a reduction that may be explained by the reduced insulin levels, as well as reduction in insulin resistance [36]. In conclusion, our study showed a statistically significant positive association of elevated level of hsCRP with T2DM. We showed a high prevalence of abnormal hsCRP among T2DM patients - as high as 27.2%. Moreover, in addition to the increased cardiovascular disease risk, hsCRP also seems to be a major inflammatory risk marker indicating renal function loss. The hsCRP test is accessible, cheap, and accurate, and one that exhibits high stability. This testing can be implemented as part of patient prognostic monitoring tools. By analyzing these readings, health professionals can recommend a set of lifestyle changes such as diet modification, weight loss, smoking cessation, and regular exercise. By optimizing CRP levels among persons diagnosed with T2DM, we can expect an overall improvement in patient condition [37]. Further studies are needed to confirm the significance of the relationship between abnormal hsCRP levels and links to cardiovascular risk and kidney dysfunction among persons with T2DM. Intervention studies targeting optimization of hsCRP levels among T2DM patients are recommended to evaluate the extent of potential benefits.

### 4.1 Limitations of the study

One limitation is that the study was conducted in a single geographic region; therefore, a long-term conclusion cannot be made and results may not apply to other geographic regions. Second, this study was a cross-sectional design, limiting ability to draw a cause- and-effect conclusion. Third, we did not measure serum insulin levels.

## Abbreviations

T2DM: type 2 diabetes mellitus

IDF: international diabetes federation

CRP: c-reactive protein

hsCRP: high sensitivity c-reactive protein

HbA1c: glycated hemoglobin

BMI: body mass index

FBS: fasting blood sugar

e-GFR: estimated glomerular filtration rate

BUN: blood urea nitrogen
